# Effects of acute exercise on spontaneous physical activity in mice at different ages

**DOI:** 10.1186/s13102-021-00311-2

**Published:** 2021-07-27

**Authors:** Ana Carolina Silvares Quintanilha, Izabelle Dias Benfato, Robson Luiz Oliveira Santos, Hanna Karen Moreira Antunes, Camila Aparecida Machado de Oliveira

**Affiliations:** Departamento de Biociências, Instituto de Saúde e Sociedade, Universidade Federal de São Paulo - UNIFESP, Campus Baixada Santista, sala 325, St. Silva Jardim 136 – Vila Mathias, SP CEP 11015-020 Santos, Brazil

**Keywords:** Energy homeostasis, Non-exercise activity, Physical activity, Ageing

## Abstract

**Background:**

Exercise is often used to obtain a negative energy balance. However, its effects on body weight reduction are usually below expectations. One possible explanation is a reduction in spontaneous physical activity (SPA) after exercise since the increase in energy expenditure caused by the exercise session would be offset by the decrease in SPA and its associated energy cost. Thus, we evaluated the effects of a single bout of moderate exercise at individualized intensity on spontaneous physical activity. The impact of the single bout of exercise was determined in early adulthood and at the transition to middle age.

**Methods:**

Male C57bl/6j (*n* = 10) mice were evaluated at 4 (4 M) and 9 (9 M) months of age. One week after a treadmill Maximal Exercise Capacity Test (MECT), mice performed a 30-min single bout of exercise at 50 % of the maximal speed reached at MECT. An infrared-based system was used to determine locomotor parameters (SPA and average speed of displacement, ASD) before (basal) and immediately after the single bout of exercise for 48 h (D1, 0-24 h; D2, 24-48 h). Food intake was measured simultaneously. Data were analyzed by GEE and statistical significance was set at *p* < 0.05.

**Results:**

Basal SPA declined from 4 M to 9 M (*p* = 0.01), but maximal exercise capacity was similar. At both ages, SPA and ASD decreased significantly (*p* < 0.0001) on day 1 after exercise. On D2, SPA returned to basal levels but ASD remained lower than basal (*p* < 0.001). The magnitude (% of basal) of change in SPA and ASD on D1 and D2 was similar at 4 M and 9 M. Food intake did not change at 4 M but decreased on D2 at 9 M.

**Conclusions:**

A single bout of moderate exercise decreases physical activity in the first 24 h and average speed of locomotion in the 48 h following exercise. This compensation is similar from early adulthood to the transition to middle age. The decrease in both the amount and intensity (speed) of SPA may compensate for the increase in energy expenditure induced by exercise, helping to understand the below-than-expected effect of exercise interventions to cause a negative energy balance.

**Supplementary Information:**

The online version contains supplementary material available at 10.1186/s13102-021-00311-2.

## New findings

**What is the central question of this study?**

Does spontaneous physical activity (SPA) decrease in response to an acute bout of moderate exercise at individualized intensity and does this compensation change as mice age from early adulthood to the transition to middle age?

**What is the main finding and its importance?**

- SPA, but not exercise capacity, declines from early adulthood to the transition to middle age;
- Acute exercise decreases SPA quantity and intensity in the following 24-48 h;
- The magnitude of the fall in SPA is similar from early adulthood to the transition to middle age;
- The fall in SPA can contribute to both body mass increase as a result of aging and to the below-than-expected effects of exercise on energy balance.

## Background

Physical exercise is widely used as a strategy to induce body weight loss. However, its effects on energy balance are commonly disappointing due to a processes referred to as compensation [1]. Among the mechanisms which may be involved in the exercise below-than-expected energy deficit are increased energy intake [1] and decreased energy expenditure, including decreased basal metabolic rate and spontaneous physical activity [2, 3].

Spontaneous physical activity (SPA) consists of daily life activities excluding structured, purposeful exercise. It generally comprises low-intensity physical activities (LIPA), with low energetic costs [4]. Recent studies showed that LIPA is associated with higher total brain volume [5] and inversely associated with all-cause mortality [6], highlighting the importance of SPA on health. The contributions of SPA to energy balance, however, are less clear, despite studies demonstrating protective effects against weight gain in response to overfeeding [7] and an inverse correlation with the rate of body weight change [8].

In humans, there is no consensus on the role of SPA in the compensation in response to exercise [1]. In rodents, the results are more consistent. We [9] and others [10–12] have shown that exercise decreases SPA. Lark et al. [12] found that this reduction was predicted to attenuate the expected change in energy balance by approximately 45 %. All of the above studies, however, used running wheel as a model of exercise, which does not allow for the control of either volume or intensity of exercise.

To gain further insight into the effects of exercise on compensation we have investigated in mice the effects of an acute session of moderate exercise at individualized intensity on 48 h SPA (amount and intensity) and energy intake, simultaneously. Moreover, as we have previously demonstrated, SPA decreases from early adulthood to the transition to middle age [13], so we also investigated whether compensation in either SPA or energy intake following exercise changes with increasing age.

## Methods

### Experimental design

 C57Bl/6j male mice (*n* = 10), obtained from the Center for Development of Experimental Models (CEDEME), Federal University of São Paulo (UNIFESP), were first acclimatized to the animal house of the Department of Bioscience. They were evaluated when they were 4 (4 M) and 9 (9 M) -month-old. A separated set of mice (*n* = 8) was used to perform a control experiment, as explained below. According to Flurkey et al. [14], this lifespan corresponds to the beginning of adult life to the transition to middle age.

### Food intake

Food intake was determined individually in the 48 h before and in the 48 h immediately after the acute exercise session, simultaneously with the measurement of SPA. Daily food consumption was determined by subtracting the weight of the remaining food after 24 h from the weight of food given, with care taken to account for spillage. Before each acute exercise session (basal) food intake was calculated as the mean of the two 24 h periods whereas food intake after the exercise was separated in day 1 and day 2.

### Maximal exercise capacity test

Mice were familiarized with the treadmill for five days before each maximal exercise capacity test (MECT), which was performed at 4 and 9 months. The acclimatization consisted of placing the mice for 30 min on a static treadmill on day 1 and, in the following days, the speed was set at 8 cm/s, which is a very low speed aimed to make mice familiar with any noise a turned-on treadmill can produce. On days 4 and 5 the mice spent an additional 10 min in the treadmill at 12 cm/s.

For the MECT, mice were placed in the treadmill for an 8-min warm-up at 12 cm/s. The test started immediately after the warm-up with an initial speed of 15 cm/s increased by 15 cm/s every 45 s until mice were not able to keep the pace even after gentle prodding by hand (adapted from [15]). The maximal speed (cm/s) at which mice completed the 45 s stage (maximal stage completed) was used to calculate the speed for the acute exercise session. The total distance (cm) and the maximal speed reached (not necessarily completing the 45 s stage) were also recorded. No electrical shock was applied for either the MECT or the acute exercise session.

### Acute exercise session

The acute exercise session was done one week after the MECT. It was performed for 30 min at 50 % of the maximal speed (cm/s) at which mice completed the 45 s stage (adapted from de [16]). When necessary, mice were gently prodded by hand. Two mice at 4 and 9 months of age were not included in the posterior analysis of spontaneous physical activity and food intake for either refusing to run for the stipulated time or for getting a small injury in the finger during the session, avoiding any interference on locomotor parameters. As a control experiment, a separated set of mice was placed in the treadmill for the same 30 min but without exercising (non-moving treadmill). They were submitted to the same locomotor (SPA and speed of displacement) and food ingestion analysis, before and after exposure to the treadmill. Additionally, on a different occasion, mice were placed in the actimeter and analyzed for 5 consecutive and uninterrupted days.

### Spontaneous Physical Activity (SPA)

Activity was measured individually by infrared beam sensors using an IR actimeter system composed of a 2 dimensional (X and Y axes) square frame (25 × 25 cm), each frame containing 16 × 16 infrared beams separated 1.3 cm from each other (Panlab-Harvard Apparatus, Barcelona, Spain). SPA was recorded in the 48 h before and in the 48 h immediately after the acute exercise session at 4 and 9 months of age. The software ActiTrack v2.7 (Panlab-Harvard Apparatus, Barcelona, Spain) also generated data on average speed of displacement. Mice were allowed 2 h of acclimatization in the actimeter cage before basal measurements. SPA consisted of the sum of stereotypes and locomotion. The software determines stereotypes by the number of samples where the position of the mouse is different from its position during the previous sample and equal to its position from the second sample, back in time; and locomotion by the number of samples where the position of the mouse is different from its position during the previous sample and different to the position of the second sample, back in time. Average speed of displacement (cm/s) is calculated as locomotion (expressed in centimeter instead of counts) divided by the time mice spent in locomotion. For basal SPA and average speed, we calculated the mean of the two 24 h periods (48 h) before the acute exercise session. After exercise, the 48-h analysis was separated in day 1 and day 2.

### Statistics

Results are presented as mean and standard deviation (SD). To evaluate the effect either age (4 and 9 months) or exercise (basal, day 1 and day 2 post-exercise), data were analyzed using Generalized Estimating Equations (GEE) on SPSS, version 20 (IBM). A pairwise Bonferroni comparison was used if necessary. The probability distribution and correlation matrix structure were chosen based on the Quasi Likelihood under Independence Model Criterion (QIC). A significance level of *p* < 0.05 was adopted.

## Results

The results of the maximal exercise capacity test are shown in Table 1. No effect of age was observed for any of the parameters evaluated at 4 and 9 months.

**Table 1:** Maximal exercise capacity test and acute exercise session at different ages

		4 M	9 M
***Maximal exercise capacity test***			
Distance (cm)		546.0 ± 105.1	603.0 ± 128.8
Total time (s)		168.7 ± 19.3	168.6 ± 20.3
Maximal speed reached (cm/s)		55.5 ± 10.1	58.5 ± 8.5
Maximal stage completed (cm/s)		52.5 ± 7.9	54.0 ± 7.7
***Acute session***			
Speed (cm/s)		26.5 ± 3.7	27.0 ± 3.9

Results are mean ± SD; Mice (*n* = 10) were followed from 4 to 9 months of age

**Fig. 1:**
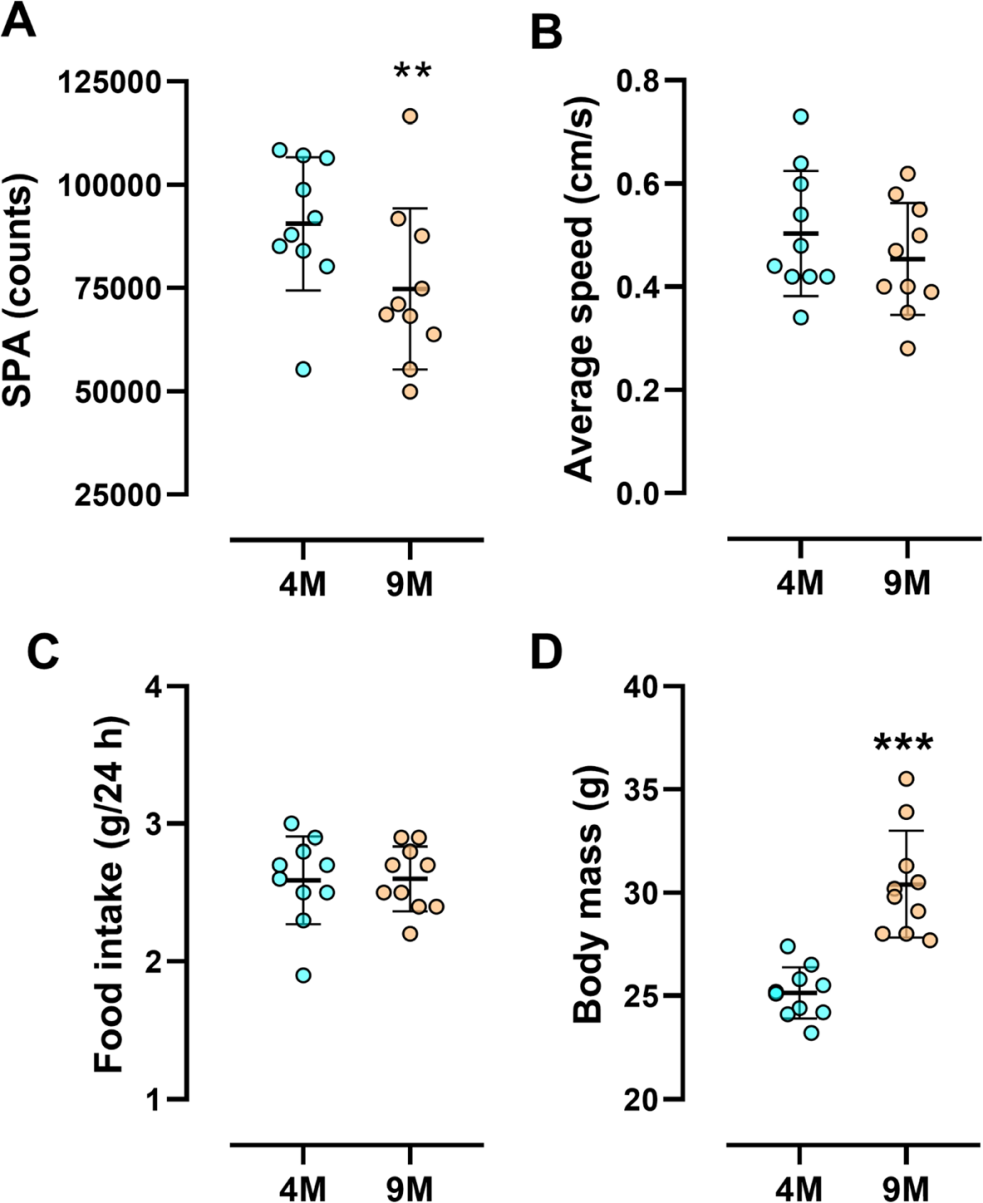
Spontaneous physical activity, average speed, food intake, and body mass through the time. Basal spontaneous physical activity (**A**), average speed of locomotion (**B**), food intake (**C**), and body mass (**D**) at different ages. Results are mean ± SD. **p < 0.01; ***p < 0.001

Figure 1 shows the effects of aging on SPA, average speed of displacement, food intake, and body mass. There was a significant effect of age on SPA (*p* = 0.01), which reduced significantly at 9M compared to 4M (Fig. 1A). Average speed of displacement did not differ significantly in the timespan studied (Fig. 1B). Food intake was also similar from 4 to 9 months of age (Fig. 1C). Body mass increased significantly (*p* = 0.0001) from 4M to 9M (Fig. 1D).

A separated set of mice was submitted to the same evaluations, but without exercising. Instead, they remained for the same 30-min in the non-moving treadmill. No difference in either SPA, speed of displacement, or food intake was observed before compared to after exposure to the treadmill (Supplementary Fig. 1A-C). In an additional analysis, mice remained for 5 consecutive and uninterrupted days in the actimeter and again, no difference was observed for either locomotor parameters or food intake (Supplementary Fig. 1D-F).

**Fig. 2:**
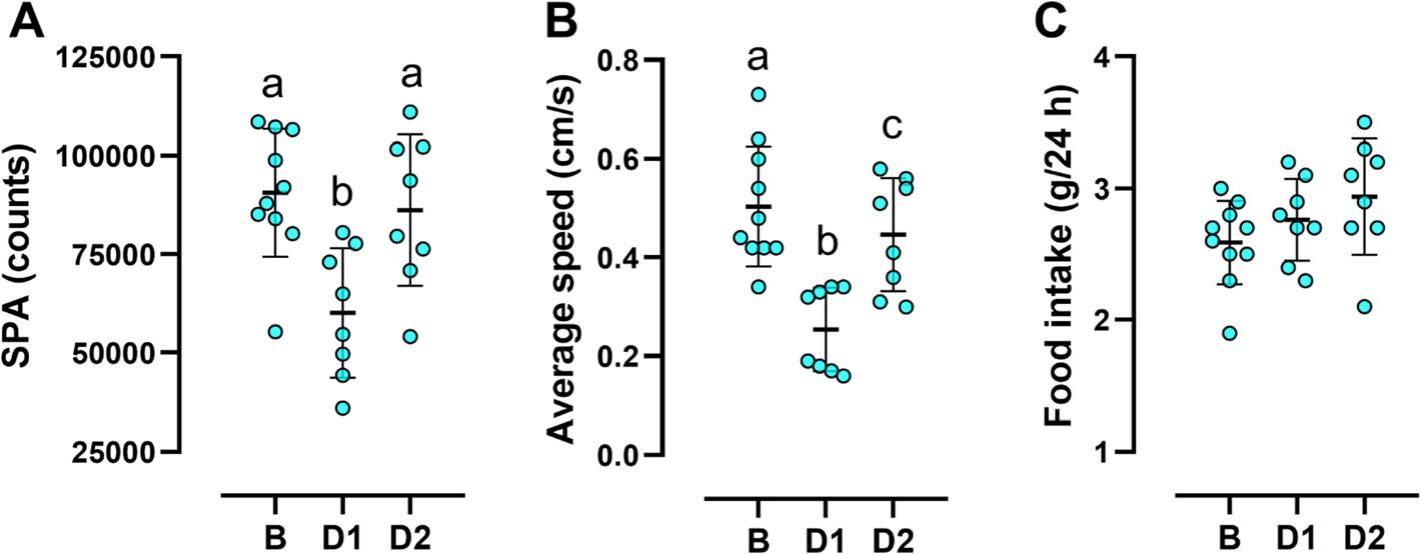
Spontaneous physical activity, average speed and food intake at 4 months of age. Spontaneous physical activity (**A**), average speed of locomotion (**B**), and food intake (**C**) in 4-month-old mice before and immediately after the acute exercise session. Results are mean ± SD; different letters indicate significant statistical difference, *p* < 0.05

The effects of an acute exercise session on SPA, average speed of displacement, and food intake in 4-month-old mice are shown in Fig. 2. There was a significant effect of exercise on SPA (*p* = 0.0001). Exercise decreased SPA (*p* = 0.0001) in the 24 h immediately after the acute session (day 1). On day 2, SPA returned to pre-exercise levels (Fig. 2A). Exercise also had a significant effect on average speed of displacement (*p* = 0.0001). The speed dropped significantly on day 1 (*p* = 0.0001) and despite increasing toward basal levels on day 2, it was still lower (*p* = 0.036) than before exercise (Fig. 2B). For food intake, despite GEE analysis indicated a significant effect of exercise (*p* = 0.041), no difference among the times (basal, day 1, and day 2) was found in the pairwise comparisons (Fig. 2C).

**Fig. 3:**
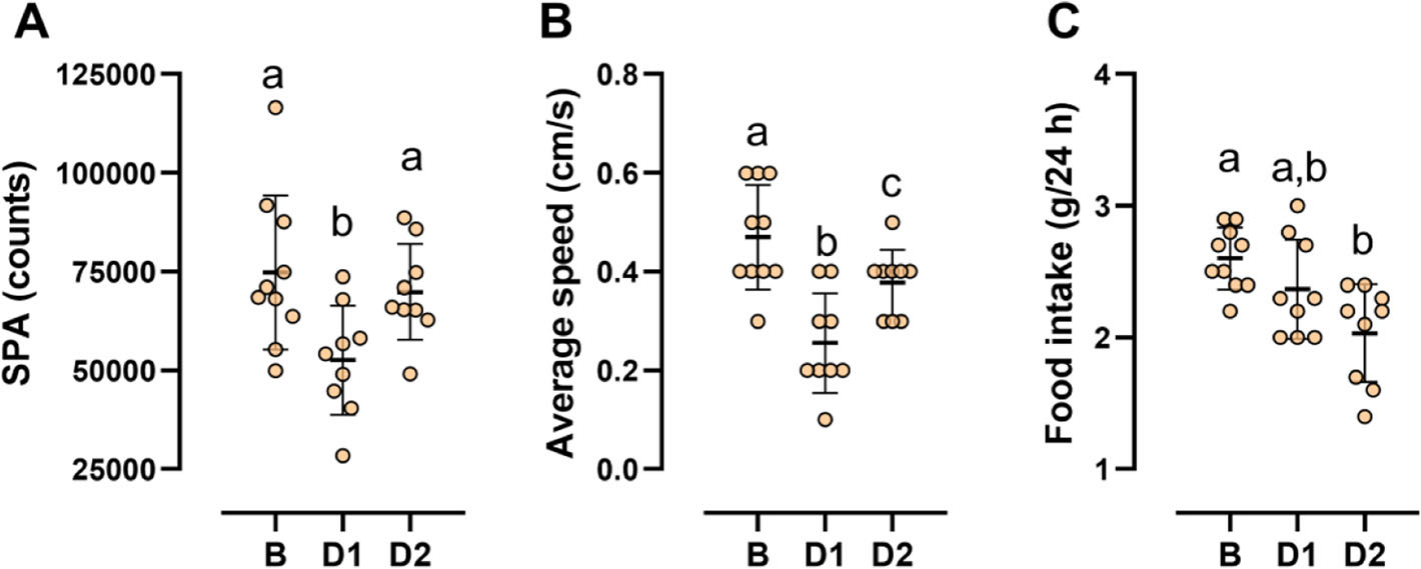
Spontaneous physical activity, average speed and food intake at 9 months of age. Spontaneous physical activity (**A**), average speed of locomotion (**B**) and food intake (**C**) in 9-month-old mice before and immediately after the acute exercise session. Results are mean ± SD; *n* = 10. Different letters indicate significant statistical difference, *p* < 0.05

As in the younger age, the acute exercise in nine-month-old mice significantly affected SPA (*p* = 0.0001). SPA reduced significantly on day 1 compared to basal (*p* = 0.0001) but was similar to pre-exercise levels on day 2 (Fig. 3A). Likewise, there was a significant effect of exercise on average speed of displacement (*p* = 0.0001). The speed fell significantly on day 1 in relation to basal (*p* = 0.0001) but, even though it increased on day 2, the average speed was still significantly lower (*p* = 0.001) than before exercise (Fig. 3B). Exercise also had a significant effect on food intake (*p* = 0.0001) but with a different temporal pattern. Compared to basal, food intake was not different on day 1 but decreased significantly (*p* = 0.0001) on day 2 (Fig. 3C).

**Fig. 4:**
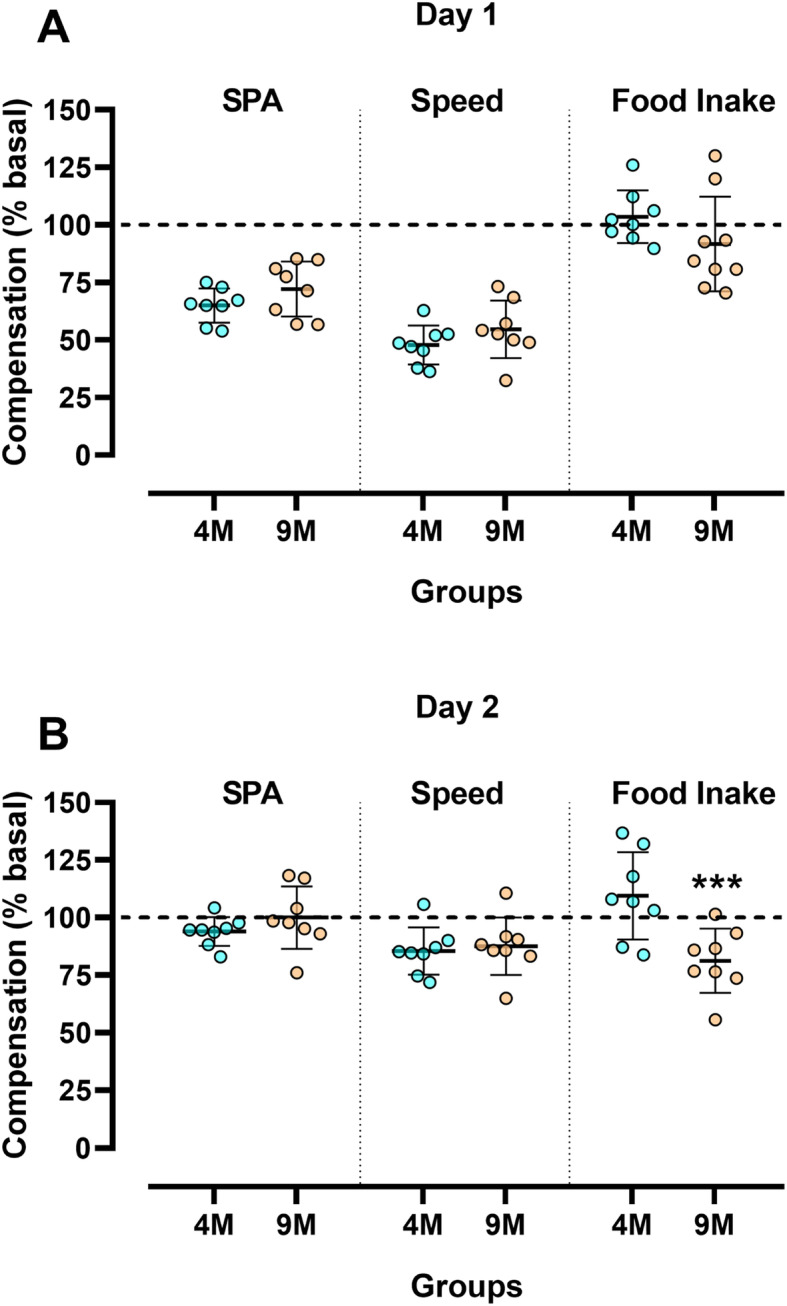
Magnitude of changes in the parameters studied at 4 and 9 months of age. Magnitude of change in spontaneous physical activity, average speed of locomotion, and food intake in the first (**A**) and second (**B**) day immediately after the acute exercise session at the different ages. Results are mean ± SD. ***p < 0.001

Next, we determined whether the magnitude of changes (% in relation to basal) in SPA, average speed of locomotion, and food intake in response to exercise was affected by age. There was no effect of age on the magnitude of change for SPA and average speed of locomotion either on day 1 (Fig. 4A) or day 2 (Fig. 4B) after exercise. For the magnitude of food intake change, there was an effect of age in response to acute exercise only on day 2 (*p* = 0.001). Whereas food consumption increased to values higher than pre-exercise at 4 M, it decreased at 9 M (Fig. 4B).

## Discussion

In our study, we found that an acute exercise session at individualized intensity consistently decreased both SPA and average speed of displacement and that the magnitude of the fall did not increase from young adulthood to the transition to middle age. Moreover, we showed that food intake was not a significant contributor to exercise compensation, favoring a role for reduced activity amount (SPA) and intensity (speed of displacement) in decrease exercise effectiveness to cause a negative energy balance.

Analyzing separately the effects of aging, we found a decrease in SPA at 9 months, as previously observed by our group [13]. During the same period, no change in absolute food intake (basal) was observed. Body mass, however, increased from 4 to 9 months of age. Different mechanisms participate in body mass gain as age increases, including decreased activity of the somatotropic axis which begins as early as after puberty, increased fat to lean muscle mass ratio [17], and reduction in resting energy expenditure that cannot be fully explained by changes in body composition [18]. Remarkably, the latter was found to be more pronounced at early adulthood in men [18]. Importantly, physical activity is a critical contributor to total daily energy expenditure [19]. Bjursell et al. [20] showed that exposure to a high-fat diet led to increased body weight gain throughout the 21 days of experiment. However, while energy intake was higher only in the first 24 h, locomotor activity reduced as early as after 3–5 h and remained lower all over, being the most important factor for body weight gain induced by the diet. Thus, taking into account that SPA comprehends all mice movement in the cage, its relevance on energy balance becomes more evident as well as the role of the decreased SPA on body mass gain with age.

The results of the maximal exercise capacity test indicate that mice at the transition to middle age have similar exercise capacity than at the beginning of adult life. SPA, as previously said, was lower at 9 M compared to 4 M, thus, suggesting that, at least in mice, the mechanism controlling SPA is more sensitive to aging and declines earlier than the function of cardiorespiratory/musculoskeletal systems. Scariot et al. [21] found that both SPA and exercise capacity declined from 2 to 5-month-old Wistar rats. Swimming exercise at individualized intensity for 12 weeks, though, avoided the decrease in SPA but not in aerobic and anaerobic capacity. Despite the differences between ours and Scariot et al. [21] study, which include distinct animals, age, exercise type, and method of SPA measurement, they both have in common the finding that SPA and exercise capacity seems to be uncoupled.

The inherent drive for movement responsible for SPA is controlled centrally and several structures and neurobiological modulators have been proposed to its regulation [22]. In a previous study, we found that leptin signaling was decreased in hypothalamus by the time SPA first started to fall, at 6 months [13]. How exercise modulates the circuitry leading to a compensatory decrease in SPA, though, needs further investigation. Exercise can, directly and indirectly, be sensed by the brain. Peripheral factors released from skeletal muscle, known as myokines, could mediate this muscle-brain crosstalk [23]. Pontzer et al. [24] proposed the “constrained-energy expenditure” theory, in which physical activity does not increase total energy expenditure in a dose-dependent manner, as an evolved mechanism to maintain energy expenditure within a physiological narrow range. In line with this theory, some myokines and other exercise mediators change in plasma according to the acute metabolic stress induced by exercise [23], which could signal to the central nervous system to decrease other energy-demanding behavior/function in order to minimize the impact on energy balance.

Different than expected, the compensation in either locomotion parameters or food intake was not more pronounced when mice were older. In humans, a compensatory decrease in SPA in response to exercise training has been observed in individuals with 56 years or more [25, 26]. However, in these studies, the results were not compared with a younger group. We did not find any study performed with rodents comparing the effects of exercise on SPA at different stages of life. Concerning food intake, a minor to lack of effects of an acute exercise session is commonly observed in humans, as reviewed by Caudwell et al. [27]. In mice, contrasting results can be found, probably, due to varying volumes of exercise and duration of the energy consumption measurement [28, 29]. Nevertheless, in Wistar rats, as they aged from 4 to 11 and from 25 to 27 months, there was an increase in the effects of appetite-suppressing factors and a decrease in the appetite-stimulating factors [30]. If similar alterations take place in mice, it could help to explain the reduction in food intake in 9-month-old mice 2 days after the acute exercise session.

A limitation of our study is that we evaluated SPA only in response to a single bout of exercise, and not after training. A compensatory decrease in cage activity has been seen in mice even after 10 weeks of wheel-running [9], implying that exercise training might not mitigate this behavior. Other authors also reported decreased SPA after chronic 3 to 8 weeks-exposure to running wheel [10, 11]. However, whereas with running wheel mice are free to exercise as they will, our study advances as our protocol allowed for the control of both volume and intensity of exercise, which are key parameters for exercise prescription. Also, it would be interesting to evaluate the same compensatory behaviors in obese mice, since they would benefit the most from negative energy balance.

## Conclusions

**Fig. 5:**
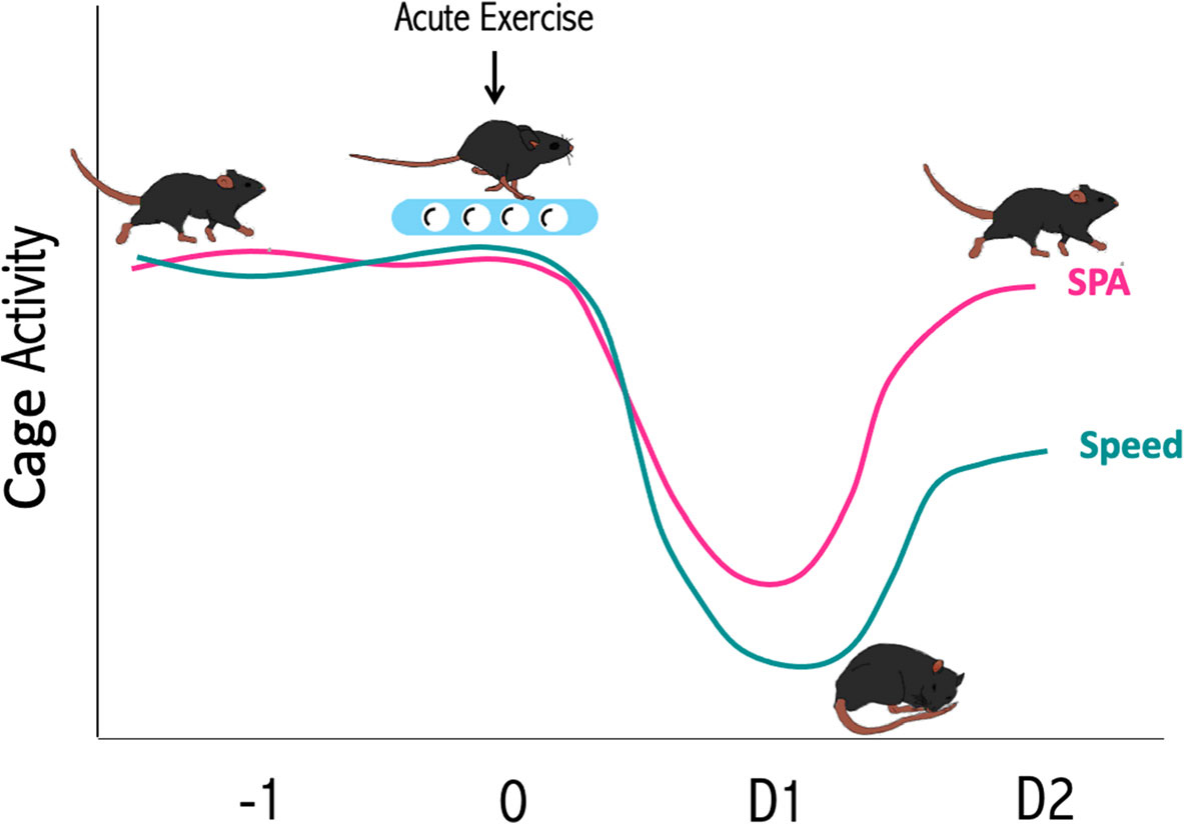
Schematic representation of the main results. Acute exercise reduces spontaneous physical activity for 24 h (day 1) and average speed of locomotion for 48 h (days 1 and 2). The effect is the same at 4 or 9 months of age

In conclusion, as summarized in Fig. 5, a single bout of moderate exercise decreases spontaneous physical activity amount and intensity in the subsequent 24-48 h with minor effects on energy intake. The magnitude of this compensatory reduction in SPA is similar from early adulthood to the transition to middle age. The decrease in both the amount and intensity of SPA may compensate for the increase in energy expenditure induced by exercise, helping to understand the below-than-expected effect of exercise interventions in causing a negative energy balance.

## Supplementary Information

**Additional file 1: Supp. Fig. 1. **Spontaneous physical activity, average speed of locomotion and food intake in mice before and after 30-min in a non-moving treadmill (A-C) or evaluated for five consecutive and uninterrupted days (D-F). Results are mean + SD; *n*=8.

## Data Availability

The datasets used and/or analyzed during the current study are available from the corresponding author on reasonable request.

## Abbreviations

ASD: Average speed of displacement
GEE: Generalizing estimating equation
LIPA: Low intensity physical activity
MECT: Maximal exercise capacity test
QIC: Quasi likelihood under independence model criterion
SPA: Spontaneous physical activity
