# Supplementary material for: Effects of acute exercise on spontaneous physical activity in mice at different ages
Source: BMC Sports Sci Med Rehabil. 2021 Jul 27;13:78. doi: 10.1186/s13102-021-00311-2 (PMC8317422; doi:10.1186/s13102-021-00311-2)
Supplement: Supplementary file 1 — Additional file 1: Supp. Fig. 1. Spontaneous physical activity, average speed of locomotion and food intake in mice before and after 30-min in a non-moving treadmill (A-C) or evaluated for five consecutive and uninterrupted days (D-F). Results are mean + SD; n=8. [file 13102_2021_311_MOESM1_ESM.docx]

**Supplementary Figure**

**Supplementary Figure 1. Spontaneous physical activity, average speed of locomotion and food intake in mice not exercised.**


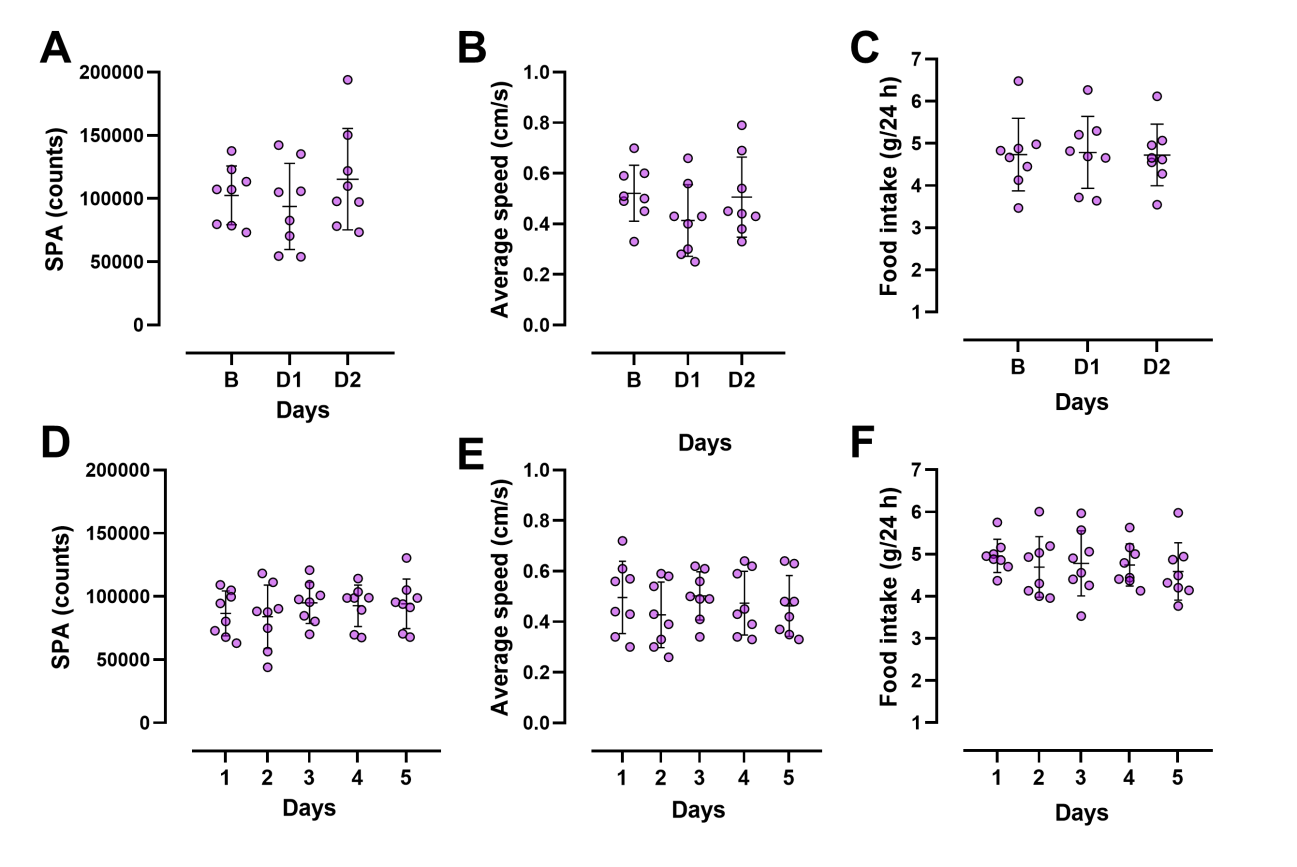


**Supp. Fig. 1. Spontaneous physical activity, average speed of locomotion and food intake in mice before and after 30-min in a non-moving treadmill (A-C) or evaluated for five consecutive and uninterrupted days (D-F).** Results are mean + SD**; n=8.**
